# Does Early Victimization of School Bullies Affect Core Self-Evaluations in Young Adulthood? A Moderated Mediation Model

**DOI:** 10.3390/ijerph20043338

**Published:** 2023-02-14

**Authors:** Lixia Wang, Shuzhi Zhou, Yukang Xue, Qianqian Li, Min Cao, Chuanhua Gu

**Affiliations:** 1 School of Psychology, Central China Normal University, Wuhan 430079, China; 2 Mental Health Education Center, Xinyang Normal University, Xinyang 464000, China; 3 Mental Health Education Center, Wuhan University of Technology, Wuhan 430070, China; 4 Department of Educational and Counseling Psychology, University at Albany, Albany, NY 12222, USA

**Keywords:** victimization, negative cognitive processing bias, resilience, core self-evaluations

## Abstract

Early victimization is associated with a range of psychological adaptation problems in young adulthood, including core self-evaluations. However, little is known about the mechanisms underlying the association between early victimization and young adults’ core self-evaluations. This study examined the mediating role of negative cognitive processing bias and the moderating role of resilience in the relationship. A total of 972 college students were recruited to complete measures of early victimization, negative cognitive processing bias, resilience, and core self-evaluations. The results showed that early victimization significantly and negatively predicted the core self-evaluations in young adulthood. The negative association between early victimization and core self-evaluations was completely mediated by negative cognitive processing bias. Resilience moderated the relationship between early victimization and negative cognitive bias, and the relationship between negative cognitive processing bias and core self-evaluations. Resilience has both risk-buffering and risk-enhancing effects. In light of these results, in order to help victims maintain good mental health, we should intervene in individual cognitive factors. Notably, while resilience is a protective factor in most cases, the benefits of resilience should not be overstated. So, we should not only cultivate students’ resilience but also provide them with more support and resources and intervene in risk factors at the same time.

## 1. Introduction

Core self-evaluations refer to the most basic evaluation and estimation of individuals’ self-ability and value [1]. It comprises self-esteem, generalized self-efficacy, locus of control, and emotional stability (low neuroticism) [2]. As a positive self-concept, core self-evaluations are associated with a range of positive outcomes [3,4,5]. The development of an overall and consistent self-concept (core self-evaluations) is a key stage-salient task in young adulthood [6]. Young adulthood is a transitional period between adolescence and adulthood; in this period, individual self-consciousness develops significantly, but it is easy to be influenced by others. Research showed that core self-evaluations will fluctuate over time due to changes in perceptions of social value and acceptance in interpersonal relationships [7]. Good core self-evaluations are of great significance to college students’ academic and healthy development [8]. Thus, exploring the potential factors that may affect college students’ core self-evaluations, and developing targeted intervention measures to effectively improve core self-evaluations is conducive to the healthy growth of college students.

### 1.1. Victimization and Core Self-Evaluations

Bullying is usually defined as “a subset of aggressive behavior characterized by repetition and an imbalance of power” [9]. It has a pronounced impact on individuals’ physical and mental health and safety [10]. Researchers defined different participant roles in bullying, including bullies, reinforcers (who encourage the bully or laugh at the victim), defenders (who help the victim), bystanders, and the victims [11]. Victimization has always been a serious social issue of great concern [12], and how victimization impacts feelings about self-worth has also attracted the attention of researchers [13]. Although negative self-evaluation is closely related to its status in peer communication, it is more likely to be the result of being bullied [14].

Victimization happens when a student is exposed, repeatedly and over time, to one or more negative actions from other students [15]. Being bullied has a negative impact on the social, academic, physical, and mental health of children and adolescents [16,17,18]. Previous research found that peer victimization can decrease core self-evaluations [19,20]. There are several reasons supporting the notion that early victimization may affect young adults’ core self-evaluations. Firstly, according to Eriksson’s theory of development [6] and attachment theory [21], peer relationships have a great impact on the self-development of individuals in primary and secondary schools. When students feel excluded, rejected, and lack a sense of belonging to a group, they will experience a decline in self-esteem and perceive themselves negatively [22], while exclusion and rejection are the basic components of bullying [20]. Secondly, studies found that peer victimization can decline students’ self-confidence, self-esteem, and self-efficacy and increase their generally negative evaluation of themselves [23,24]. Moreover, victimization is widespread among children and adolescents [25], while the negative effects of early victimization will persist into adulthood [13,16,17,26,27,28,29]. For instance, Blood and Blood [16] found that adults who suffered childhood victimization reported low levels of self-esteem. deLara [17] found in a qualitative study that young adults (18–29 years old) who suffered peer victimization in grades Kindergarten to 12 in the U.S. showed low levels of self-esteem and high levels of shame (which led them to question themselves: “Am I no good?”). These studies both show that childhood victimization has been tied to lower and decreasing self-esteem in adulthood [13]. To sum up, we put forward the following hypothesis:

**Hypothesis 1** **(H1):** *Early victimization (in primary and secondary schools) would affect the core self-evaluations in young adulthood.*

However, the current research on bullying mainly focuses on children or adolescents and studies the immediate or contemporaneous consequences of these adverse experiences [17]. There is still a lack of research on how early victimization experiences affect self-development in young adulthood, yet it is necessary [13,17]. The organism–environment interaction model points out that individuals’ attitudes and behaviors are the result of the interaction between individual factors and environmental factors [30]. Specifically, to fill these gaps, the present study examined the impact of contextual factors (i.e., early victimization) and personal factors (i.e., negative cognitive processing bias and resilience) on young adults’ core self-evaluations. The findings would advance our understanding of the mechanisms underpinning the association between early victimization and young adults’ core self-evaluations, provide effective intervention on the negative effects of school bullying, and effectively improve college students’ core self-evaluations.

### 1.2. The Mediating Role of Negative Cognitive Processing Bias

Negative cognitive processing bias is a cognitive style with which individuals have a preference for processing negative information, and it includes four common forms: negative attention bias, negative memory bias, negative interpretation bias, and negative rumination bias [31]. A developmental model of negative cognition bias proposed by Beck [32] focused on the emergence and later impact of negative cognition after negative events such as trauma and bullying. It suggested that early adverse events could lead to the formation of negative schemas, which could be activated later in life by an event or a series of events resulting in negative cognitive bias. Consistent with Beck’s theory, victimization, as an extremely difficult life experience [13], can also lead to negative cognition or schemas [33,34]. Research showed that peer victimization can make individuals form abnormal schema and negative thinking [35]. At the same time, it will lead to the development of negative schema, dysfunctional cognition, and negative cognitive bias [36].

While one underlying driver leading to low self-evaluations is the way in which individuals interpret their experiences of victimization [13]. Negative cognitive processing bias is the automatic processing of negative information [37], showing negative views about the self [31]. According to attribution theory [38], victims with negative cognitive processing bias will show more negative internal attribution, such as self-blame, believing that they are flawed. They tend to interpret and internalize bullying into negative beliefs, think they are losers, unwelcome, and unpleasant, and hold negative evaluations of themselves [15,39]. In addition, they often internalize their companions’ ridicule and taunt to form a negative self-impression [40]. Thus, victims with negative cognitive processing bias have more negative core self-evaluations.

Early victimization can lead to one or more victim responses and consequences in young adulthood through individual cognition [17]. Similarly, McDougall and Vaillancourt [13] proposed that cognition is one factor predicting whether early peer victimization might continue into adulthood. Specifically, according to negative cognitive bias theory [32] and attribution theory [38], early victimization will lead to negative cognitive processing bias, while individuals with negative cognitive processing bias tend to carry on negative information processing on victimization experiences, which leads to the decreases of core self-evaluations. Meanwhile, it is of great significance to explore the mediating role of negative cognitive processing bias because cognition is relatively plastic and the negative cognition of the victims can be rectified by cognitive restructuring intervention [41], thereby reducing the negative effect of victimization on core self-evaluations. Overall, we put forward the following hypothesis:

**Hypothesis 2** **(H2):** *Negative cognitive processing bias would mediate the relationship between early victimization and young adulthood core self-evaluations.*

### 1.3. The Moderating Role of Resilience

Although early victimization can have a negative impact on individuals’ physical and mental health, not all bullied individuals will be equally affected [26]. The theory of organism–environment interaction [30] points out that individuals in the same or similar environments may have different reactions and performances due to their different traits. Resilience is an individual trait often considered when studying the heterogeneity in people’s responses to all manner of environmental adversities [42]. Resilience refers to an individual’s ability to successfully cope with negative events [43]. High levels of resilience are related to many positive outcomes [44,45]. In addition, resilience also has a positive association with self-esteem (the core form of core self-evaluations). Aliyev and Gengec [46] found that internet bullying can reduce self-esteem, while resilience can improve self-esteem. Resilience is a protective factor that buffers the negative impact of victimization [47,48]. Moreover, many empirical studies have found that resilience also moderated the relationship between victimization and negative outcomes [47,49,50]. For example, Moore and Woodcock [47] found that those with higher levels of resilience were less affected by victimization in terms of their psychosocial adaptation and subjective well-being. This may be because individuals with high levels of resilience have the determination to face life’s challenges, have self-confidence, and can successfully deal with these challenges [51]. They are also more able to maintain positive emotions and cognitions [52]. In addition, students with high levels of resilience are less likely to report that they had been bullied at school or online, and even if they were bullied, resilience seemed to serve as a protective factor, insulating them from the negative effects caused by victimization [53].

To our knowledge, no research to date has examined whether early victimization interacts with resilience to predict negative cognitive processing bias or core self-evaluations. Students with high levels of resilience have more positive cognition and self-evaluation [42], so they can actively deal with victimization and reduce the negative impact of victimization. Thus, resilience may moderate the association between early victimization and negative cognitive processing bias and core self-evaluations. Moreover, students with negative cognitive processing bias may be protected by their resilience, which enables them to recover from negative cognitive bias quickly and achieve positive results (e.g., positive core self-evaluations). Thus, resilience may be a protective factor that reduces the impact of negative cognitive processing bias on core self-evaluations. Overall, we put forward the following hypotheses:

**Hypothesis 3** **(H3):** *Resilience would moderate the relation between early victimization and core self-evaluations, with the relation being stronger for students with lower resilience.*

**Hypothesis 4** **(H4):** *Resilience would moderate the mediating effect of negative cognitive processing bias in the relation between early victimization and core self-evaluations, with the meditating effect of negative cognitive processing bias being stronger for students with lower resilience.*

### 1.4. The Current Study

**Figure 1:**
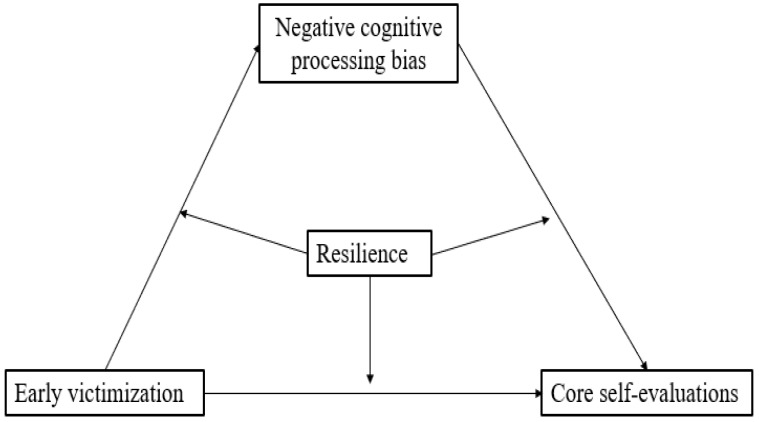
The conceptual model.

In summary, based on Erikson and Erikson’s theory of development [6], the developmental model of negative cognition bias [32], organism–environment interaction theory [30], and prior studies, the present study examined how early victimization affects core self-evaluations (important for young adults’ development) in young adulthood. Specially, we examined a moderated mediation model (see Figure 1) to explore the impact of contextual factors (i.e., early victimization) and personal factors (i.e., negative cognitive processing bias and resilience) on young adults’ core self-evaluations. Providing empirical evidence for the long-term effect of early adverse experiences on self-development and practical guidance for intervention programs.

## 2. Methods

### 2.1. Participants and Procedure

A total of 972 college students (40% were males) from three universities in central China completed our survey that was designed to collect information including demographic variables, early victimization, core self-evaluations, negative cognitive processing bias, and resilience. The mean age of the participants was 19.25 years old (SD = 1.17, range = 16–25). There were 388 freshmen, 279 sophomores, 295 juniors, and 10 missing data.

We recruited participants through a random cluster sampling method. Specifically, we chose three classes in each grade, from freshman to junior, from the target universities. Students in target classes were invited to participate in the survey anonymously in classrooms. Students took about 15 min to complete all the questionnaires. All measures were administered by trained graduate students in psychology and headteachers using standardized instructions. This study was reviewed and approved by the Research Ethics Committee of the author’s institution, and all participants provided informed consent.

### 2.2. Measures

Early victimization. Early victimization was assessed by the Chinese version of the junior version of the Olweus Bully/Victim Questionnaire [54]. Six items related to verbal, relational, and physical victimization were used to measure participants’ past victimization experience in primary and secondary schools (e.g., “Some classmates gave me bad names to scold me, or made fun of me and satirized me”). The instruction was set to “Please recall if you had any of the following experiences in primary and secondary school”. Participants rated these items on a four-point scale (1 = never, 4 = always). Average scores of all items were computed, with higher scores indicating higher frequencies of early victimization. In the present study, Cronbach’s *α* for the questionnaire was 0.84.

Negative cognitive processing bias. The negative cognitive processing bias was measured by the Negative Cognitive Processing Bias Questionnaire developed by Yan et al. [31]. Participants answered 23 items assessing four dimensions of negative cognitive processing bias: negative attention bias, negative memory bias, negative interpretation bias, and negative rumination bias (e.g., “I vividly remember a time when I was laughed at”). Each item was answered on a four-point scale (1 = not at all true, 4 = completely true). Average scores of all items were computed, with higher scores representing a higher tendency for negative cognitive processing bias. In the present study, Cronbach’s *α* for the questionnaire was 0.89.

Resilience. Resilience was assessed using the Resilient Trait Scale for Chinese Adults [55]. The scale includes 30 items measuring five dimensions of resilience: internal locus of control, problem-focused coping style, optimism, predisposition to accepting and utilizing social supports, and acceptance (e.g., “I can overcome difficulties mainly because of my strong ability”). Participants rated their perceptions of resilience on a four-point scale (1 = not at all true, 4 = completely true). Average scores of all items were computed, with higher scores reflecting higher levels of resilience. In the present study, Cronbach’s *α* for the questionnaire was 0.91.

Core self-evaluations. Core self-evaluations were measured by the Chinese version of the Core Self-Evaluations Scale (CSES) [56,57]. The CSES consists of 10 items (e.g., “I am confident I can succeed in life”). Each item was rated on a five-point scale (1 = strongly disagree, 5 = strongly agree). Average scores of all items were computed, with higher scores indicating higher levels of core self-evaluations. In the present study, Cronbach’s *α* for the questionnaire was 0.86.

### 2.3. Statistical Analysis

Three major steps were conducted for data analyses. Firstly, Harman’s single-factor test [58] was used to check for common-method variance as all data in this study was gathered through the same method, self-report. Secondly, descriptive statistics and correlation analyses were conducted using SPSS 22.0. Prior research suggested that gender has an impact on victimization [59]. Thus, we controlled for gender in our statistical analyses. Thirdly, we examined the moderated mediation model using the SPSS macro PROCESS (model 59) suggested by Hayes [60]. The SPSS macro PROCESS is specifically developed for testing complex models that include both mediator and moderator variables, and it has been used by lots of scholars. One advantage of PROCESS is that it automatically generates bootstrap confidence intervals, which explain the possible non-normality of the sampling distribution [22]. The bootstrap method, based on 5000 resamples to date, produces 95% bias-corrected confidence intervals. To intuitively show the moderation effect, the interaction effects were plotted with one standard deviation above and below the mean of resilience.

## 3. Results

### 3.1. Common-Method Bias Test

Since the data were all collected from self-reported questionnaires, common-method bias may exist. According to the suggestion from Zhou and Long [61], both procedural control and statistical control were carried out to eliminate common-method bias. In terms of procedural control, we tried our best to change instructions and scoring methods when designing the questionnaires. Some items were scored in reverse, and the participants filled in the questionnaires anonymously. As for statistical control, Harman’s single-factor test [58] was used to test the common-method variance. All the items in the study were entered into an exploratory factor analysis. The results revealed 13 factors with eigenvalues greater than 1.0. Moreover, the first factor accounted for 20.07% of the variance, which was no more than 40%, indicating the common-method bias was not a concern in this study [62].

### 3.2. Preliminary Analyses

Descriptive statistics and Pearson product-moment correlation coefficients for study variables are presented in Table 1. Early victimization was positively correlated with negative cognitive processing bias, and negatively correlated with resilience and core self-evaluations. Negative cognitive processing bias was negatively correlated with resilience and core self-evaluations. Resilience was positively correlated with core self-evaluations.

### 3.3. Testing for the Moderated Mediation Model

We used Hayes’ SPSS macro PROCESS (model 59) [60] to test the moderated mediation model. Specifically, we estimated the parameters for two regression models. In model 1, we estimated the moderating role of resilience in the relationship between early victimization and negative cognitive processing bias. In model 2, we estimated the moderating role of resilience in the relationship between negative cognitive processing bias and core self-evaluations, as well as the direct relationships between early victimization and core self-evaluations. The main results are presented in Table 2.

As shown in Table 2, after controlling for gender, model 1 indicated that early victimization positively predicted negative cognitive processing bias (*β* = 0.28, *t* = 8.30, *p* < 0.001), the interaction of early victimization and resilience significantly predicted negative cognitive processing bias (*β* = 0.10, *t* = 2.93, *p* < 0.01). Model 2 indicated that early victimization did not significantly predict core self-evaluations (*β* = −0.04, *t* = −1.33, *p* = 0.18), and the interaction of early victimization and resilience also had no significant predictive effect on core self-evaluations (*β* = −0.03, *t* = −0.91, *p* = 0.37). Negative cognitive processing bias significantly and negatively predicted core self-evaluations (*β* = −0.27, *t* = −10.10, *p* < 0.001), the interaction of negative cognitive processing bias and resilience also significantly predicted core self-evaluations (*β* = 0.08, *t* = 3.92, *p* < 0.001).

**Figure 2:**
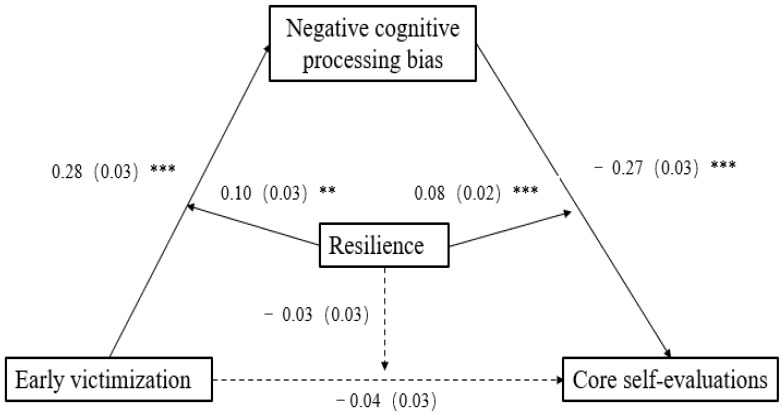
The moderated mediation model. Negative cognitive processing bias completely mediated the relationship between early victimization and core self-evaluation. Resilience moderated the first and second parts of the mediation process. Path values are the path coefficients (standard errors). (The dotted line indicates that *p* is greater than 0.05). *** *p* < 0.001. ** *p* < 0.01.

**Figure 3:**
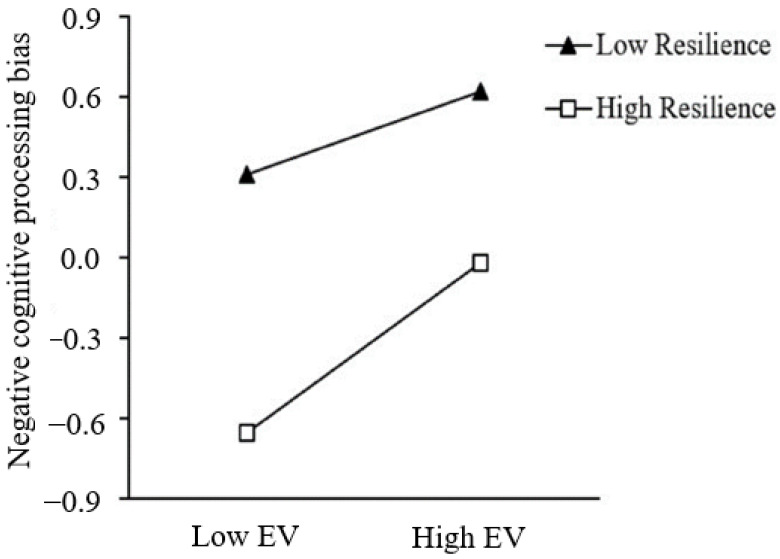
Resilience moderates the relation between early victimization and negative cognitive processing bias.

**Figure 4:**
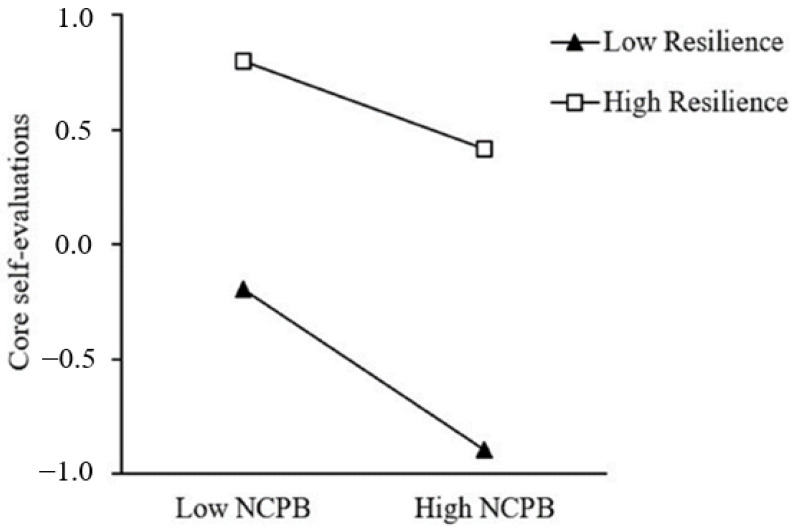
Resilience moderates the relation between negative cognitive processing bias and core self-evaluations.

Based on these results, both H1 and H2 are supported. Specifically, negative cognitive processing bias completely mediated the relationship between early victimization and core self-evaluations. The conditional indirect effect was −0.06 at −1 standard deviation and −0.07 at +1 standard deviation. Resilience moderated the first and second parts of the mediation process. While the direct relationships between early victimization and core self-evaluations were not moderated by resilience (see Figure 2). Simple slope analyses were then conducted to help better understand the interaction effect (see Figure 3 and Figure 4). As shown in Figure 2, the adverse impact of early victimization on negative cognitive processing bias was stronger when resilience was high (1 SD above the mean), relative to low (1 SD below the mean) (*β_high_* = 0.38, *p* < 0.001; *β_low_* = 0.18, *p* < 0.001). While in Figure 3, the adverse impact of negative cognitive processing bias on core self-evaluations was weaker when resilience was high, relative to low (*β_high_* = −0.19, *p* < 0.001; *β_low_* = −0.35, *p* < 0.001). Furthermore, as can be seen from the conditional indirect effect analysis, the indirect effect of early victimization on core self-evaluations was stronger for the high levels of resilience group (*β* = −0.07, *p* < 0.001) than for the low levels of resilience (*β* = −0.06, *p* < 0.001). Therefore, H4 was partially supported.

## 4. Discussion

The present study indicated that early victimization could negatively predict core self-evaluations among young adults. It further confirmed that early victimization could have long-term negative impacts on individuals’ self-evaluation [13,16,63]. Besides, this study constructed a moderated mediation model to examine how early victimization influences core self-evaluations, and whether young adults with different resilience levels are equally influenced by early victimization. We found that negative cognitive processing bias completely mediated the relation between early victimization and core self-evaluations, and this mediating process was moderated by resilience. Those findings are compatible with the developmental model of negative cognition bias proposed by Beck [32], suggesting that early victimization can cause negative schemas, in turn resulting in negative self-evaluation. And the outcome of individual psychological development is the result of the interaction between environmental (early victimization) and individual factors (resilience) [30]. Moreover, the moderated mediation model also sheds light on the mechanisms and conditions through which early victimization decreases young adults’ core self-evaluations. The findings are of great significance to deepening the theoretical research of school bullying, as well as guiding interventions on the mental health of bully victims.

Importantly, our study indicated that negative cognitive processing bias completely mediated the relationship between early victimization and core self-evaluations. External information and life events can only have an impact on an individual’s self-evaluations through the subjective cognition of the individual. Previous studies have revealed that negative cognition is at the core of the development and maintenance of post-traumatic mental health symptoms [64]. As for this study, the experience of being bullied does not necessarily lead to a decrease in core self-evaluations. Only when individuals developed a negative cognitive processing bias due to past bullying experiences did they tend to have a negative self-evaluation. Early victimization is an important negative event for students [65]. The results of this study indicated that college students who have been bullied in primary/secondary school are more likely to hold negative cognition. The result is consistent with previous research indicating that individuals who have been bullied tend to have negative schemas [32] and interpret this experience as the result of negative peer evaluation or social exclusion [66]. Furthermore, this study also found that negative cognitive bias resulted in negative self-evaluations. Consistent with the prior result that individuals who experience peer victimization and rejection are more likely to hold a negative view of themselves, which makes them feel inadequate in their social skills and ability to get along with their peers, and has lower self-efficacy [67] and self-esteem [68], and finally reinforces their negative self-evaluations.

This study showed that resilience moderated the first (i.e., the impact of early victimization on negative cognitive processing bias) and the second part of the mediation process (i.e., the impact of negative cognitive processing bias on core self-evaluations). There are different theoretical explanations when considering the moderating role of resilience. The risk-buffering model proposes that high personal assets (i.e., resilience) attenuate the negative association between risk factors and capacity development [69]. According to this model, the higher the levels of resilience, the weaker the harmful impact of early victimization. In contrast, the risk-enhancing model argues that once environmental risks are too serious, personal assets may lose their ability to counteract risks. That is, the protective effects of personal assets are weakened in the face of high environmental risks [69]. In this case, the high levels of resilience alone cannot reduce the adverse impact of early victimization. On the one hand, consistent with the risk-buffering model, resilience, as a positive factor, may attenuate the negative impact of early victimization on negative cognitive processing bias and core self-evaluations. On the other hand, early victimization may have particularly harmful and long-term impacts on individuals [68]. At the same time, some researchers have begun to pay attention to the possible side effects of resilience [49,70]. Thus, the risk-enhancing model may be applicable.

Specifically, in the first part, although resilience played a protective role at low levels of victimization, with the increases in early victimization, its benefits were gradually weakened, and the positive relationship between early victimization and negative cognitive processing bias was stronger among students with high resilience. This was more parallel to the risk-enhancing model. The following reasons may explain it. First, First, individuals with high levels of resilience tend to have better peer relationships and social skills [41], and they are less likely to be the target of bullying [53]. Therefore, this contradictory state will lead to self-doubt and cognitive bias in people with high resilience. Second, it is possible that individuals with high levels of resilience overly rely on their personal assets to counteract bullying; thus, extreme resilience may drive them to be overly persistent in their attempt to overcome bullying [49]. While the efforts may be associated with negative outcomes in situations where individuals have less control, as in the case of bullying. Besides, high levels of resilience are associated with being overly tolerant and indulgent of bullying, which leads to the increase or persistence of victimization and hence a faster depletion of coping resources [49], resulting in more negative outcomes (i.e., negative cognitive processing bias). Besides, according to Erikson and Erikson’s [6] theory of development, peer relationships are one of the most important relationships for individuals in primary and secondary school. Being rejected or bullied by peers is a highly influential negative event for individuals [71] and casts a long shadow over their lives [72]. Thus, resilience alone cannot offset the negative effects. As research showed, when victims are rejected in some contexts, maternal and sibling warmth and a positive family atmosphere are negatively associated with emotional and behavioral problems within two years of being bullied [73]. Accordingly, for the victims, good relationship support may be more helpful in mitigating the negative effects of being bullied than their own resilience. Therefore, future studies could examine whether cumulative protective factors from different sources can act as risk buffer factors, which can also help to design more comprehensive intervention programs.

However, in the second part of the mediating pathway, our results showed that resilience played a role in risk-buffering. For individuals with negative cognitive processing bias, those with higher levels of resilience can still maintain good core self-evaluations. The negative relation between negative cognitive processing bias and core self-evaluations was weaker among students with high levels of resilience. This is consistent with previous research showing that people with high levels of resilience have higher cognitive abilities and are less likely to interpret traumatic experiences as their own fault [42]. Besides, individuals with high levels of resilience are not inclined to regard harmful behavior as intentional, nor are they inclined to regard it as bullying. Therefore, their self-evaluations are not always determined by a negative cognitive processing bias. However, individuals with low levels of resilience may have formed a negative cognitive processing schema in their daily lives. When the schema is activated by an event or a series of events, it will distort the information processing system, making individuals pay attention to negative stimuli and draw negative interpretations of the experiences [32]. Thus, the core self-evaluations of individuals with low levels of resilience are more susceptible to negative cognitive processing bias.

In summary, this study advanced the understanding of the impacts of early victimization on young adults’ core self-evaluations. The findings also demonstrate the necessity of cognitive modification in reducing the negative effect of early victimization on core self-evaluations and the potential mixed role of resilience, which provides evidence for further intervention. In practice, first, we should be alert to the long-term negative effects of early victimization. Second, the results suggest that we can make intervention programs aimed at cognitive restructuring for victims. Third, considering the risk-buffering and risk-enhancing roles of resilience, we can improve college students’ resilience, for it is a protective factor against the negative effects of risks in most cases, but the benefits of resilience should not be overstated, especially when students encounter major adversity, which they cannot cope with well by themselves. At this time, we should not only cultivate their resilience but also provide them with more support and resources and intervene in risk factors (i.e., timely detection of bullying and timely prevention of it).

### Limitations and Future Directions

This study has several limitations. First, only self-report questionnaires were used in this study. College students’ reports of early victimization experiences were retrospective; they may be affected by social desirability and recall bias. Data from other sources, such as parents, teachers, and the participants’ companions, could be collected in future studies to confirm the results of this study. Second, we cannot make causal inferences about the results based on the cross-sectional design used in the present study. For example, it could be low-core self-evaluations that lead to victimization. One prior study found that self-esteem, as an important facet of core self-evaluations, has a bidirectional relationship with victimization [74]. Therefore, longitudinal designs can be adopted to examine the bidirectional effect between bullying experience and core self-evaluations in future research. Third, this study only considers traditional bullying and does not measure the increasingly prevalent online bullying in the digital environment [75]. Future research can focus on individual mental health and its mechanisms in the context of cyberbullying. Finally, our study found that resilience did not play a buffering role in the relationship between negative cognitive processing bias and early victimization. Future research needs to further examine when resilience works as a protective factor and when it does not. Moreover, other protective factors, such as social support, a positive family atmosphere, and peer warmth, should be considered, and their cumulative protective effects along with resilience could be explored in future research.

## 5. Conclusions

This study constructed a moderated mediation model to examine the mediating role of negative cognitive processing bias and the moderating role of resilience. The findings suggest that early bullying victimization was significantly and negatively related to core self-evaluations in young adulthood. Negative cognitive processing bias completely mediated the relation between early bullying victimization and core self-evaluations. Resilience moderated the indirect pathways from early bullying victimization to young adults’ core self-evaluations. The results also remind us that although resilience is a protective factor, its benefits should not be overstated, especially in cases of high levels of bullying victimization.

## Figures and Tables

**Table 1:** Descriptive statistics and correlations for study variables (N = 972).

	M	SD	1	2	3	4
1 EBV	1.29	0.41	1			
2 NCPB	2.21	0.48	0.30 **	1		
3 Resilience	2.82	0.38	−0.27 **	−0.48 **	1	
4 CSE	3.51	0.64	−0.21 **	−0.56 **	0.69 **	1

Note: EBV = early victimization, NCPB = negative cognitive processing bias, CSE = core self-evaluations; ** *p* < 0.01.

**Table 2:** Testing the moderated mediation model.

Model	Predictors	*β*	*SE*	*t*	95%CI
Model 1 (NCPB)	Gender	0.28	0.06	4.77 ***	[0.17, 0.40]
	EV	0.28	0.03	8.30 ***	[0.21, 0.35]
	Resilience	−0.42	0.03	−14.23 ***	[−0.48, −0.36]
	EV × Resilience	0.10	0.03	2.93 **	[0.03, 0.16]
	R^2^	0.29			
	F	91.83 ***			
Model 2 (CSE)	Gender	−0.22	0.05	−4.55 ***	[−0.31, −0.13]
	EV	−0.04	0.03	−1.33	[−0.09, 0.02]
	Resilience	0.58	0.03	21.75 ***	[0.53, 0.64]
	EV × Resilience	−0.03	0.03	−0.91	[−0.08, 0.03]
	NCPB	−0.27	0.03	−10.10 ***	[−0.32, −0.22]
	NCPB × Resilience	0.08	0.02	3.92 ***	[0.04, 0.12]
	R^2^	0.56			
	F	184.79 ***			
Conditional direct effect analysis at Resilience = M ± SD		*β*	Boot SE	BootLLCI	BootULCI
M − 1 SD (2.44)		−0.01	0.03	−0.07	0.05
M (2.82)		−0.04	0.03	−0.09	0.02
M + 1 SD (3.20)		−0.06	0.05	−0.16	0.03
Conditional indirect effect analysis at Resilience = M ± SD		*β*	Boot SE	BootLLCI	BootULCI
M − 1 SD (2.44)		−0.06	0.02	−0.10	−0.03
M (2.82)		−0.08	0.01	−0.10	−0.05
M + 1 SD (3.20)		−0.07	0.02	−0.11	−0.04

Note: NCPB = negative cognitive processing bias, EV = early victimization, CSE = core self-evaluations; LL = low limit, CI = confidence interval, UL = upper limit, M − 1 SD (2.44) means 1 SD below the mean and M + 1 SD (3.20) means 1 SD above the mean; *** *p* < 0.001. ** *p* < 0.01.

## Data Availability

The data can be obtained from the corresponding author for justified reasons.
